# A Comprehensive Review of One-Dimensional Metal-Oxide Nanostructure Photodetectors

**DOI:** 10.3390/s90806504

**Published:** 2009-08-20

**Authors:** Tianyou Zhai, Xiaosheng Fang, Meiyong Liao, Xijin Xu, Haibo Zeng, Bando Yoshio, Dmitri Golberg

**Affiliations:** 1 World Premier International Center for Materials Nanoarchitectonics (MANA), National Institute for Materials Science (NIMS), Namiki 1-1, Tsukuba, Ibaraki 305-0044, Japan; 2 Sensor Materials Center, National Institute for Materials Science (NIMS), Namiki 1-1, Tsukuba, Ibaraki 305-0044, Japan

**Keywords:** metal oxide semiconductor, one-dimensional nanostructures, sensor, photodetector, transistor

## Abstract

One-dimensional (1D) metal-oxide nanostructures are ideal systems for exploring a large number of novel phenomena at the nanoscale and investigating size and dimensionality dependence of nanostructure properties for potential applications. The construction and integration of photodetectors or optical switches based on such nanostructures with tailored geometries have rapidly advanced in recent years. Active 1D nanostructure photodetector elements can be configured either as resistors whose conductions are altered by a charge-transfer process or as field-effect transistors (FET) whose properties can be controlled by applying appropriate potentials onto the gates. Functionalizing the structure surfaces offers another avenue for expanding the sensor capabilities. This article provides a comprehensive review on the state-of-the-art research activities in the photodetector field. It mainly focuses on the metal oxide 1D nanostructures such as ZnO, SnO_2_, Cu_2_O, Ga_2_O_3_, Fe_2_O_3_, In_2_O_3_, CdO, CeO_2_, and their photoresponses. The review begins with a survey of quasi 1D metal-oxide semiconductor nanostructures and the photodetector principle, then shows the recent progresses on several kinds of important metal-oxide nanostructures and their photoresponses and briefly presents some additional prospective metal-oxide 1D nanomaterials. Finally, the review is concluded with some perspectives and outlook on the future developments in this area.

## 1. Introduction

One-dimensional (1D) nanomaterials have stimulated great interest due to their importance in basic scientific research and potential technological applications [1–3]. It is generally accepted that 1D nanostructures are ideal systems for exploring a large number of novel phenomena at the nanoscale and investigating the size and dimensionality dependence of structure properties for potential applications [4]. 1D nanomaterials are also expected to play an important role as both interconnects and functional units in fabricating electronic, optoelectronic, electrochemical, and electromechanical devices with nanoscale dimensions [5]. Among the inorganic semiconductor nanomaterials, 1D metal oxide nanostructures are the focus of current research efforts in nanotechnology since they are the most common minerals on the Earth due to their special shapes, compositions, and chemical, and physical properties. They have now been widely used in many areas, such as transparent electronics, piezoelectric transducers, ceramics, catalysis, sensors, electro-optical and electro-chromic devices [6–8]. Doubtlessly, a thorough understanding of the fundamental properties of a 1D metal oxide system is prerequisite in research and development towards practical applications.

Table 1 shows the fundamental physical properties of some important metal-oxide semiconductors, including zinc oxide (ZnO), tin dioxide (SnO_2_), copper oxide (Cu_2_O), Gallium oxide (*β-*Ga_2_O_3_), Hematite (α-Fe_2_O_3_), Indium oxide (In_2_O_3_), Cadmium oxide (CdO) and Ceria (CeO_2_).

Among all nanoscale devices, the photodetectors are critical for applications as binary switches in imaging techniques and light-wave communications, as well as in future memory storage and optoelectronic circuits [10,11]. With large surface-to-volume ratios and Debye length comparable to their small size, these 1D nanostructures have already displayed superior sensitivity to light in experimental devices. Until now, various 1D metal-oxide nanostructures have been used for the fabrication of photodetectors, and the related mechanism has been investigated over the years. This mechanism is outlined below, using ZnO nanowire (NW) as a model system. Essentially, all experiments carried out to date on metal-oxide nanostructures indicated that the role of oxygen vacancies is predominant for the electronic properties, similar to the bulk systems [8].

**Figure 1.**
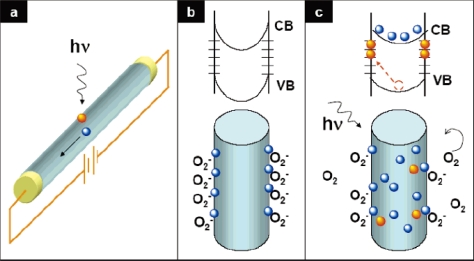
Photoconduction in NW photodetectors: (a) Schematic of a NW photodetector. Upon illumination with the photo-energy above Eg, electron-hole pairs are generated and holes are readily trapped at the surface. Under an applied electric field, the unpaired electrons are collected at the anode, which leads to the increase in conductivity. (b, c) Trapping and photoconduction mechanism in zinc oxide (ZnO) NWs: the top drawing in (b) shows the schematic of the energy band diagrams of a ZnO NW in dark, indicating band-bending and surface trap states. VB and CB are the valence and conduction bands, respectively. The bottom drawing shows oxygen molecules absorbed at the NW surface that capture the free electrons present in the n-type semiconductor forming a low-conductivity depletion layer near the surface. (c) Under light illumination, photogenerated holes migrate to the surface and become trapped, leaving behind unpaired electrons in the NW that contribute to the photocurrent. In ZnO NWs, the lifetime of the unpaired electrons is further increased by oxygen molecules desorption from the surface when holes neutralize the oxygen ions. Reproduced from [9].

As discussed by Yang *et al*, Wang *et al* and other groups [9,12–14], in the dark, the oxygen molecules absorb on the ZnO NW surface and capture the free electrons present in a n-type oxide semiconductor [ $O_{2}\;(g)+e^{-}\to O_{2}^{-}\;(\mathrm{ad})$], and a low-conductivity depletion layer is formed near the surface (Figure 1b), that results in the reduction of the channel conduction. When the ZnO NW is illuminated by a UV-light whose photon energy is above the energy gap (E_g_) of the ZnO, then the electron-hole pairs are photogenerated [*hν*→*e*^−^ + *h*^+^], and the holes migrate to the surface along the potential slope produced by band bending and discharge the negatively charged absorbed oxygen ions through surface electron-hole recombination [ $h^{+}+O_{2}^{-}\;(\mathrm{ad})\to O_{2}\;(g)$]. Consequently, oxygen is photodesorbed from the surface. The unpaired electrons are either collected at the anode or recombine with holes generated when oxygen molecules are reabsorbed and ionized at the surface. The hole-trapping mechanism through oxygen desorption in ZnO NWs augments the high density of trap states (usually found in NWs) due to the dangling bonds at the surface, and thus greatly increases the NW photoconductivity [9]. With respect to a traditional film photodetector, 1D metal-oxide nanostructures have several advantages. namely a large surface-to-volume ratio with the carrier and photon confinement in two dimensions, superior stability owing to high crystallinity, possible surface functionalization with target-specific receptor species, and field-effect transistor configurations that allow the use of gate potentials controlling the sensitivity and selectivity [15].

This article provides a comprehensive review of the state-of-the-art research activities that focus on several kinds of important metal-oxide nanostructures such as ZnO, SnO_2_, Ga_2_O_3_, Cu_2_O, Fe_2_O_3_, In_2_O_3_, CdO, CeO_2_, and their corresponding photodetector applications, and briefly discusses some other metal-oxide semiconductors. In the end, we conclude this review with some perspectives/outlook and future research directions in this field.

## 2. Different Photodetector Materials—Metal Oxides

In this section, we highlight recent progresses with respect to several kinds of metal-oxide nanostructures, including ZnO, SnO_2_, Ga_2_O_3_, Fe_2_O_3_, In_2_O_3_, CdO, Cu_2_O and CeO_2_, and their photoresponses.

### 2.1. ZnO-Based Photodetectors

ZnO is one of the most prominent semiconductors in the metal-oxide family. It has a wide-band-gap of 3.37 eV and a large exciton binding energy of 60 meV. This ensures efficient excitonic ultraviolet (UV) emission at room temperature. Besides, the non-central symmetry of ZnO in wurtzite structure, combined with its large electromechanical coupling, results in strong piezoelectric and pyroelectrical properties and implies a consequent usage in actuators, piezoelectric sensors and nanogenerators. ZnO is also bio-safe, biocompatible, and can be directly used for biomedical applications without coatings [4]. As for 1D ZnO nanostructures, they play the key roles in developing nanoscience and nanotechnology, as illustrated by many articles published. It is fair to state that ZnO 1D nanostructures are probably the most important Metal-Oxide-Semiconductor 1D nanostructures in nowadays research. Growing interests in the synthesis of ZnO nanostructures are stimulated due to promising applications in nanoscale technologies and devices. ZnO diverse and versatile morphologies are probably wider than any other materials known to date [16,17].

**Figure 2.**
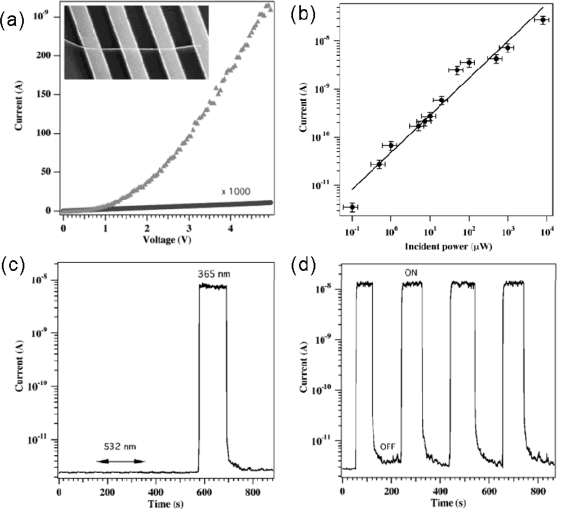
(a) Current-Voltage (I–V) curves showing dark current and photocurrent of a single ZnO NW under 365 nm UV-light illumination; (b) Photocurrent as a function of light intensity at 365 nm; (c) Sensitivity of the photoresponse of a ZnO NW to light exposure at a wavelength of 532 nm and 365 nm; (d) Current-time (I–t) plot recorded with the UV illumination turned on and off repeatedly. Reproduced from [12].

In this section, we will first give two examples related to “Nanowire Ultraviolet Photodetectors and Optical Switches”, and “Photoswitches and Memories Assembled by Electrospinning Aluminum-Doped Zinc Oxide Single Nanowires”, and then report the systematic investigations on photodetector applications based on ZnO nanostructures. In 2002, Yang and coworkers first found that the conductance of ZnO NW had been extremely sensitive to ultraviolet light exposure [12]. The light-induced conductivity enabled them to reversibly switch the NWs between OFF and ON states. In a typical experiment, four-terminal measurements on an individual ZnO structure indicated that they were almost insulating in the dark with a resistivity of ∼3.5 MΩ cm^−1^. When the NWs were exposed to 365 nm UV-light, their resistivity was remarkably reduced by 4–6 orders of magnitude, as shown in Figure 2a. Furthermore, the NW photodetector exhibited strong power dependence (Figure 2b) and excellent wavelength selectivity (Figure 2c) [12].

Yang *et al*. further evaluated the NW potential in optoelectronic switches, with the insulating state as “OFF” state in the dark, and the conductance state as “ON” when exposed to UV light. Figure 2d plots the photoresponse of a ZnO NW as a function of time while the UV-light was switched on and off. It is evident that this NW could be reversibly and rapidly switched between the low- and high-conductivity states. The rise and decay times of the fastest NW switches were below the apparatus detection limit, which was roughly 1 s [5,12].

After that, several researchers have dedicated themselves to improving the response of a ZnO 1D nanostructure photodetector, and obtained a series of remarkable achievements. He and coauthors utilized focused-ion-beam (FIB) technique to deposit Pt metal on ZnO NWs and transmission-line-method (TLM) measurements to effectively reduce the contact resistance which was as low as 1.1 × 10^5^ Ωcm^2^, and thus achieved ZnO NW-based UV photodetectors with a photoconductive gain as high as 10^8^ (the photoconductive gain is one of the most important physical parameters which determines the photocarrier collection efficiency) [13].

Prades and coauthors presented a set of criteria to optimize a ZnO NW photodetector. They enhanced its response through different fabrication strategies, such as diminishing the distance between the electrical contacts, increasing the width of the photoactive area, or improving the electrical mobility of the nanomaterials [18]. Very recently, Kim and coauthors demonstrated that drain-source (V_ds_) and gate-source voltages (V_gs_) of a ZnO NW FET could be optimized to increase UV photodetection sensitivity. They found that the detector was the most sensitive when it was operated with the highest on/off current ratio and at the “bottom” of the sub-threshold swing region. Their photodetector showed maximum photo- to dark- current ratio of ∼10^6^ upon UV illumination near V_gs_ = −1 V and V_ds_ = 1 V. These results could be applied to improve sensitivity of other kinds of FET-based photodetectors whose sensing mechanism is based on the change of carrier concentration [19].

**Figure 3.**
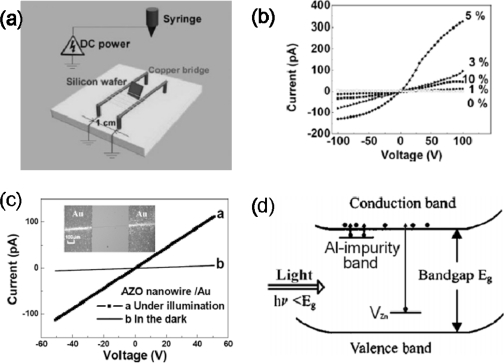
(a) Schematic illustration of the setup used for electrospinning nanowires as uniaxially aligned arrays; (b) The measured current-voltage (I–V) curves of undoped and Al-doped samples; (c) Photoresponse of the Al-doped ZnO nanowires (1 at% Al) to below-gap illumination (Au electrodes); (d) Schematic energy band diagram of the photoswitches. Reproduced from [20].

Recently, several doped 1D ZnO nanostructures have been synthesized and investigated for enhancing and controlling ZnO nanostructures’ mechanical, electrical and optical performances. Pan *et al*. reported on a photoconductor device that was sensitive to illumination with below-gap light by electrospinning a single Al doped-ZnO (AZO) nanowire. By using a “Cu bridge” as the cathode (Figure 3a), the electrospun nanowires could be uniaxially aligned over long length scales during the e-spinning process. The nanowires were then directly transferred to a SiO_2_/Si substrate, and calcined at 550 °C for 3 h to obtain well-aligned polycrystalline AZO nanowires. A typical I-V curve for a single AZO nanowire with different Al concentrations is shown in Figure 3b. Compared with the pure ZnO nanowires with a conductivity of 2.55×10^−5^ S/cm, the Al-doped ZnO nanowires show a steep increase up to 9.73 × 10^−3^ S/cm (Al = 5%), indicating a great enhancement in conductivity. Alumina acts as a cationic dopant in the ZnO lattice, that is, the trivalent Al^3+^ ion occupies the divalent Zn^2+^ site ( ${\mathrm{Al}}_{\mathrm{Zn}}^{•}$) allowing electrons to move to the conduction band easily.

Figure 3c shows the I–V curves measured in dark and under illumination for comparison. As the device was illuminated by a white-light source with ∼400–800 nm, the conductance of an individual AZO (Al ∼1.0 at %) nanowire increased significantly from 3.36 × 10^−4^ S/cm to 6.67 × 10^−3^ S/cm, which is about 20 times of that measured in darkness. The authors suggest that the notable photoresponse of the AZO nanowires may be attributed to photo-excitation of electrons from the defect levels introduced by Al doping. As light with energy below the bandgap is introduced, the electrons captured at the detect states, such as ${\mathrm{Al}}_{\mathrm{Zn}}^{•}$, $V_{\mathrm{Zn}}^{\prime \prime }$, as well as an impurity band of ca. 80 meV binding energy below the effective band edge of ZnO, are photo-excited to the conduction band, as illustrated in Figure 3d.

The increase in carrier density thus greatly enhances the conductivity of the AZO nanowire. In contrast, an extremely weak photoresponse of undoped ZnO nanowires was observed, with only a small current rise of about 6.4% [20]. Very recently, Xie *et al*. have found a negative photoconductivity of Co-doped ZnO nanobelts. The decrease of photoconductivity from 1.3 to 0.25 μA in ambient air or water vapor atmosphere is observed when the Co-doped ZnO nanobelts are irradiated with 630 nm light. The authors think that this kind of negative photoconductivity is attributed to the photodesorption of water molecules from nanobelts’ surface [28]. Meanwhile, Kouklin *et al*. reported the fabrication of Cu-doped ZnO nanowires for efficient and multispectral photodetection applications. The use of Cu is shown to dramatically enhance the photosensitivity levels of the nanowires to both UV and visible light as a result of avalanche photomultiplication [38].

It should be stated that within last few years, tremendous progress has already been made in photodetector applications of ZnO 1D nanostructures. For instance, we realized that there have been more than 50 research papers published solely related to ZnO 1D nanostructure photodetector characteristics. Here, we tabulate the representative results on photodetector properties of ZnO nanostructures reported so far, along with a brief description of the corresponding device continuations, detection wavelength, and photodetector performance (Table 2).

### 2.2. SnO_2_-Based Photodetectors

SnO_2_, as another important n-type metal-oxide semiconductor, is particularly interesting and has many important applications. Its large bandgap (E_g_ = 3.6 eV at 300 K) makes it ideally working as a transparent conducting electrode for organic light emitting diodes and solar cells [46,47]. In addition, SnO_2_ films and nanostructures have extensively been studied and used as chemical sensors for environmental and industrial applications.

**Figure 4.**
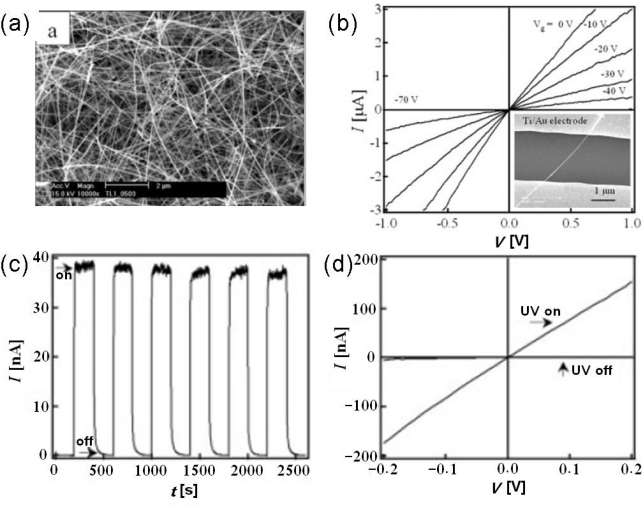
(a) Scanning electron microscope (SEM) image of SnO_2_ NWs; (b) Gate-dependent I–V curves of a single SnO_2_ NW device; (c) I–t curve recorded with the UV illumination turned on and off repeatedly; (d) I–V curves taken with and without UV illumination. Reproduced from [46].

Zhou *et al*. reported the preparation of SnO_2_ NWs using a laser-ablation technique. The NW diameters can be precisely controlled by using mono-dispersed gold clusters as the catalyst. Most of the NWs have diameters around 20 nm and lengths of the order of 10 μm, as illustrated in Figure 4a. Field-effect transistors were made based on these SnO_2_ NWs, and n-type transistor characteristics were recorded with threshold voltages of 50 V and on/off ratios of 10^3^ at room temperature. The authors also investigated the photoresponse properties of a SnO_2_ NW transistor. As shown in Figure 4c, the current increased dramatically and stabilized a high-conductivity “on” state (∼760 nS) upon UV exposure; whereas it decreased quickly and ended up at a low-conductivity “off” state (∼0.66 nS) after the UV light was blocked, leading to an on/off ratio as high as 10^3^. The response time of this device was less than 0.1 s [46]. To date, there have been only a few reports that deal with photodetector properties of 1D SnO_2_ nanostructures, as shown in Table 3.

### 2.3. Cu_2_O-based photodetectors

Copper oxide (Cu_2_O) is one of the first known p-type direct band gap semiconductors. It has the advantages of nontoxicity, low cost, availability, high absorption coefficients at 2.17 eV, and energy conversion efficiencies [49]. Its outstanding excitonic properties including a large exciton binding energy (∼150 meV) have been the target of the most of research efforts during the past decades. Due to their unique physical and chemical properties, Cu_2_O materials have found prominent applications in field-effect transistors, photovoltaic devices, sensors, and photo-electrodes in high-efficiency photo-electrochemical cells [48,50].

**Figure 5.**
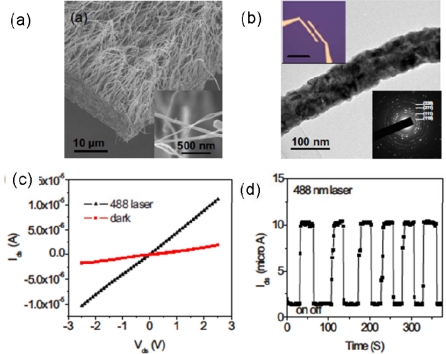
(a, b) Scanning electron microscopy (SEM) image, transmission electron microscopy (TEM) image and selected-area electron diffraction (SAED) pattern of Cu_2_O NWs; (c) I–V curves show dark current and photocurrent of a single Cu_2_O NW under 488 nm laser illumination; (d) I–t curves recorded with the 488 nm laser illumination turned on and off repeatedly. Reproduced from [48].

Yu *et al*. reported that Cu_2_O NWs could be conveniently synthesized by reduction of CuO NWs in hydrogen gas. The SEM image shows that the prepared aligned Cu_2_O NWs on the substrate are ∼20–30 μm in length and ∼50–100 nm in diameter. A typical TEM image of the Cu_2_O NWs is shown in Figure 5b. A selected area diffraction pattern of the NW reveals that it is polycrystalline. Figure 5c shows I–V characteristics of the Cu_2_O NW, measured in dark room and under blue light (488 nm) laser illumination. As known, photogenerated carriers could significantly increase the conductivity when semiconductor materials are illuminated by high energy photons. Meanwhile, the large surface to volume ratio of semiconductor NWs is able to further enhance the sensitivity of the NW device to light and even possibly lead to the realization of single photoconductivity. Thus the conductance of NW increased from 0.7 to 4.3 μS under the illumination. Time-resolved measurements of the photoresponse to a 488 nm light were conducted and the results are shown in Figure 5d. The “on” current and “off” current for each of six cycles remains the same within the noise envelope, indicating the reversibility and stability of the Cu_2_O NWs optical switches. The photoconductivity response time is less than three seconds. These results show that the Cu_2_O NW devices have a fast photoresponse to blue illumination in air and at room temperature [48].

### 2.4. Ga_2_O_3_-based photodetectors’

Monoclinic gallium oxide (*β-*Ga_2_O_3_) is a promising oxide semiconductor with a wide direct hand gap of 4.9 eV [52,53]. It is chemically and thermally stable and has been widely used as an insulating oxide layer in gallium-based electrical devices. *β-*Ga_2_O_3_ is an n-type semiconductor at elevated temperatures and its semiconductivity is governed by an oxygen deficiency of the crystal lattice [54]. With its surprising bulk properties, such as conduction and luminescence, this material can be applied for high temperature gas sensing, solar cells, flat-panel displays and optical limiters for ultraviolet irradiation [55].

**Figure 6.**
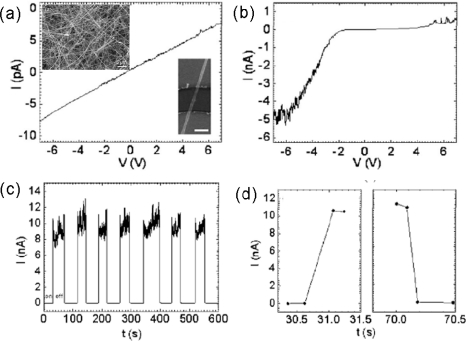
(a, b) I–V curves of a detector (a) without and (b) under 254 nm light illumination. Insets of Figure 5a are typical SEM images of *β-*Ga_2_O_3_ NWs and the device; (d) I–t curves recorded with the 254 nm light illumination turned on and off repeatedly; (e) Enlarged rise and decay edges for the first “ON” and “OFF”, respectively. Reproduced from [51].

Recently, an individual *β-*Ga_2_O_3_ NW as a solar-blind photodetector was investigated by Wang and coworkers. The SEM image depicts the general morphology of *β-*Ga_2_O_3_ NWs. They have diameters of several tens of nanometers and length of a few tens of micrometers. I–V curves of the photodetector device without and under 254 nm light illumination are shown in Figures 6a and 6b. As the device is exposed to illumination by an ultraviolet lamp, its conductance greatly increases. Due to the poor ohmic contacts between the electrodes and the NWs, the I–V curve of the device under illumination is asymmetric and nonlinear. Figure 6d shows the real-time response of the detectors to 254 nm light by ON/OFF switching at a bias of −8 V. It can be seen that the dark current is of the order of pA. Upon illumination, the current rapidly increases to several nA, but the current fluctuations are a little larger (maybe due to the surface species absorption/desorption or appearance of defects). As the light is off, the current suddenly decreases to its original value. The detectors operated with an upper limit of the response time of 0.22 s, an upper limit of the recovery time of 0.09 s, and a sensitivity of ∼1000. These results demonstrate that *β-*Ga_2_O_3_ NWs are promising materials for realizing solar-blind photodetectors [51].

### 2.5. Fe_2_O_3_-based photodetectors

Hematite (α-Fe_2_O_3_) is a stable n-type semiconductor with unique magnetic properties and thermal stability. Due to its narrow band gap of 2.1 eV, α-Fe_2_O_3_ NWs were applied in water splitting and solar cells [56].

**Figure 7.**
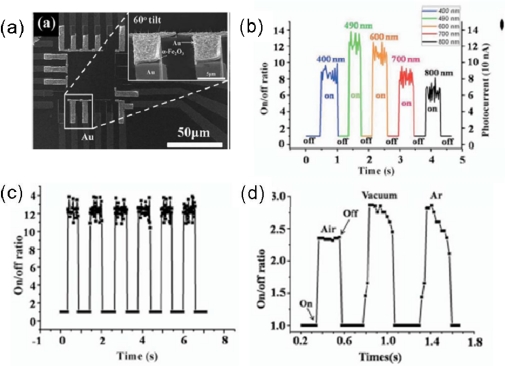
(a) SEM image of Fe_2_O_3_ nanobridge photodetectors; (b) On/off ratio as a function of time under light illumination at 400, 490, 600, 700, and 800 nm; (c) Response time of the α-Fe_2_O_3_ nanobridge to pulsed light illumination (490 nm); (d) On/off ratio as a function of time under light illumination at 420 nm in air, vacuum and Ar. Reproduced from [56].

Li *et al*. for the first time reported a visible light photodetector made of an individual α-Fe_2_O_3_ NW. The authors introduced a simple thermal oxidation process to form an individual α-Fe_2_O_3_ nanobridge (NB) photodetector with a diameter of 8 nm between the electrodes on a Si_3_N_4_/Si substrate. From the SEM (Figure 7a) and TEM images, it is understood that the NWs had been grown laterally from one side of the Fe film to another, which acted as a NB to connect the two patterns. The diameter and the length of an individual NB were ∼8 nm and ∼240 nm, respectively. HRTEM and SAED results demonstrated that the NWs are crystallized in a rhombohedral structure with the growth direction along the [110]. The photocurrent properties of an individual α-Fe_2_O_3_ NB device were then investigated in detail. The photocurrent can be enhanced by illumination at 400–800 nm (visible light). The maximum photocurrent was ∼123 nA at ∼490 nm. As shown in Figure 7b, the on/off ratios were ∼12 for 490 nm and 11 for 600 nm, and the response time for the visible light was less than 20 ms. The gain of the α-Fe_2_O_3_ NB device was calculated to be 2.9 × 10^7^. The authors also investigated the response of the on/off ratio with or without illumination in air, Ar or vacuum. The measured photocurrents and response time were both increased in oxygen-free environment (under vacuum or Ar) as shown in Figure 7d [56].

### 2.6. In_2_O_3_-Based Photodetector

**Figure 8.**
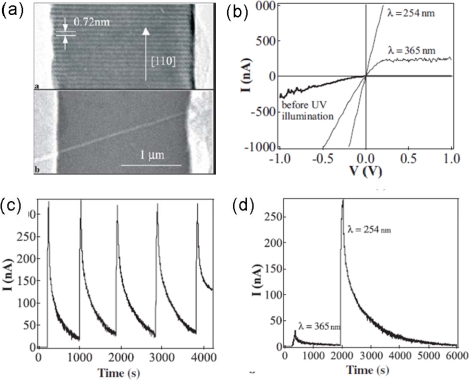
(a) HRTEM image of an In_2_O_3_ NW and SEM image of a NW device; (b) I–V curves recorded before UV light illumination and after exposure to UV light at a wavelength of 365 nm and 254 nm, respectively; (c) I–t curves recorded with the 254 nm UV light illumination turned on and off repeatedly; (d) Photoresponse of the In_2_O_3_ NW to sequential UV illumination at a wavelength of 365 nm and 254 nm. Reproduced from [57].

In_2_O_3_ is a wide-bandgap transparent semiconductor (with a direct bandgap of ∼3.6 eV and an indirect bandgap of ∼2.5 eV). In its bulk form, the material has been widely used in solar cells and organic light-emitting diodes. More importantly, In_2_O_3_ films have been demonstrated to work as ultra-sensitive toxic-gas detectors with detection levels down to 5 ppm for NO_2_, due to the surface interaction and electron transfer between NO_2_ molecules and In_2_O_3_ surface. 1D In_2_O_3_ nanostructures are expected to offer enhanced sensitivity and an improved response time due to the increased surface-to-volume ratios. Zhou’s group has conducted an excellent work on the fabrication, electronic transport and ultraviolet photodetection properties of In_2_O_3_ NWs [57–59]. The In_2_O_3_ NWs were synthesized using a laser ablation technique via the vapor-liquid-solid mechanism. The SEM and TEM results show that these NWs have well-controlled diameters of 10 nm and lengths exceeding 3 μm. The HRTEM image demonstrates that the NWs have single-crystalline structures grown along the [100] direction. Devices based on individual In_2_O_3_ NW display a substantial increase in conductance (of up to four orders of magnitude) upon exposure to UV light, as shown in Figure 8b. Figure 8d presents the time response of the device after its exposure to the 365 nm and 254 nm lights sequentially. UV light with a wavelength of 365 nm brought a current to ∼33 nA. Significantly stronger conduction (290 nA) was observed upon exposure to a 254 nm UV light. The differences in conductions are attributed to different excited models. The photo energy of 254 nm (4.9 eV) light is larger than the In_2_O_3_ direct bandgap (3.6 eV). This sufficiently excites electrons directly from the valence band to the conduction band. In contrast, the photo energy for a 365 nm light is smaller than that of In_2_O_3_ NWs but larger than the indirect energy gap of 2.5 eV. This results in the indirect transitions. Furthermore, the authors have also demonstrated that the UV light can be used as a “gas cleaner” for In_2_O_3_ NW chemical sensors, leading to a recovery time as short as 80 s [57]. However, as a photodetector, the dark decay time of this In_2_O_3_ device operated at 254 nm is a little longer.

### 2.7. CdO-Based Photodetectors

**Figure 9.**
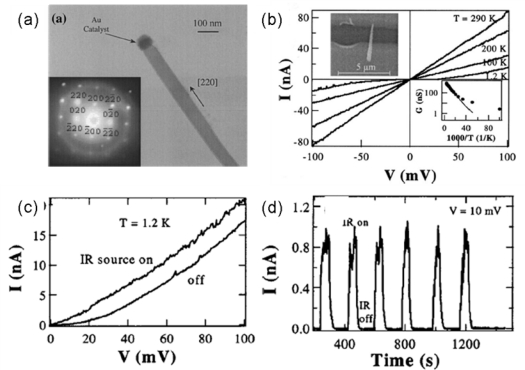
(a) TEM image and SAED pattern of a CdO nanoneedle; (b) Temperature-dependent I–V curves recorded with the temperature ranging from 290 K to 1.2 K; (c) I–V curves measured at 1.2 K with the IR light source on and off, respectively; (d) Real-time measurement of IR response of the nanoneedle device. Reproduced from [60].

Among conductive oxide (TCO) materials CdO shows a high promise. It exhibits a wide direct band gap of 2.27 eV and a narrow indirect band gap of 0.55 eV. Several techniques have been used to grow 1D CdO nanostructures, such as nanowires, nanobelts, and nanoneedles. Figure 9a shows a TEM image of a single CdO nanoneedle grown by VLS mechanism under a chemical vapor deposition process. The SAED pattern demonstrates that the CdO nanoneedles are single crystals with a cubic crystal structure grown along the [220] direction. The electrical transport and photoresponse properties were studied by fabricating electrodes onto individual nanoneedles, as illustrated in Figure 9b. Electrical properties were measured at different temperatures, revealing that the transport is dominated by thermal excitation. These CdO nanoneedle devices can absorb IR light via the indirect band gap mechanism rather than under the direct band gap process. The IR detection on/off ratio is ∼8.6 at 1.2 K and the relaxation time constant is estimated to be 8.6 s [60]. The experiments on IR detectors were carried out at 1.2 K to minimize the thermal excitation. These results indicate that these IR detectors may only work at low temperatures, not at room temperature, which will restrict its practical application.

### 2.8. CeO_2_-Based Photodetectors

**Figure 10.**
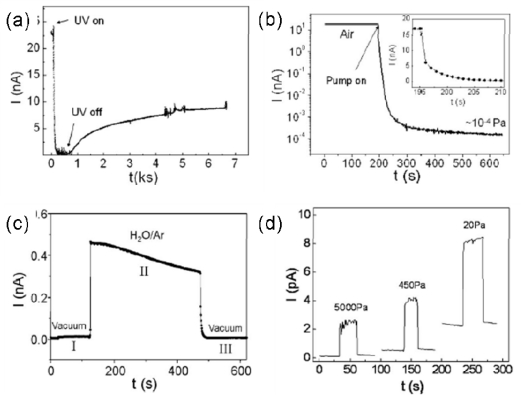
(a) UV response of CeO_2_ NWs; (b) I–t curves of the device measured in air and vacuum; (c) Photocurrent response in H_2_O/Ar mixed air; (d) UV response of CeO_2_ NWs in different dry O_2_. Reproduced from [61].

As a well-known functional rare earth material, CeO_2_ has widely been applied in many fields such as catalysts, gas sensors and optics due to its unique properties. It has strong absorption in the UV region and is used as a UV blocking and shielding material [62]. Wang *et al*. reported the fabrication of CeO_2_ NWs via a wet chemical route. These NWs, with diameters of 20–40 nm and lengths of several micrometers, were dispersed on interdigital electrodes. As shown in Figure 10a, the dark current is ∼22.8 nA in air; in contrast, upon exposure to UV illumination, the current decreases rapidly to 10 nA and then gradually decreases to 0.25 nA in 300 s, which is different from the traditional photodetectors. The authors also investigated the photoresponse behavior of the device in vacuum, H_2_O/Ar mixed gas and different dry O_2_ pressure conditions, as depicted in Figures 10c and 10d. As the pressure decreases (air to vacuum), the current decreases by nearly five orders of magnitude, from 17.1 to 1.6 × 10^−4^ nA. When H_2_O/Ar mixed gas and O_2_ are introduced into the chamber, the photocurrent increases rapidly. The authors suggested that this anomalous behavior, i.e., negative CeO_2_ NWs photoconductivity in air, was due to UV-induced desorption of H_2_O from the nanowire’s surface [61]. In regard of the photodetector application, the on-off ratio of the CeO_2_ device is a little lower and the response time is a little longer.

### 2.9. Ternary Oxide-Based Photodetectors

#### 2.9.1. ZnSnO_3_-based photodetectors

Yue *et al*. reported the photoconducting properties of individual ZnSnO_3_ NWs by performing transport measurements under UV and green laser illumination on/off circles [63]. For their experiments, the ZnSnO_3_ NWs were synthesized by thermal evaporation of ZnO, SnO and graphite mixed powders. Such NWs possessed core-shell structures. Upon exposure to UV illumination, the current of an individual ZnSnO_3_ NW increases by about three orders of magnitude, from 0.3 nA to 162 nA within 20 s. And the current increases to 6.6 nA in 20 s under green laser illumination. Such behavior was ascribed to a fact that the illuminations adjusted the grain boundary barrier (that between the core and the shell) [63].

#### 2.9.2. ZnGa_2_O_4_-based photodetectors

Zinc gallate (ZnGa_2_O_4_), with a band gap of 4.4–4.7 eV, is potentially of great technological interest for the development of luminescence, vacuum electronics and multicolor emitting phosphors. The material has received a significant attention [64]. Feng *et al*. fabricated ZnGa_2_O_4_ NWs via a low-pressure chemical vapor deposition method using Ga metal and ZnO nanopowders as the sources and investigated the electrical transport. The NWs have diameters of several tens of nanometers and lengths of a few tens of micrometers. It was found that the current across individual NW was several pA at a bias of 30 V, and the current was sensitive to oxygen and temperature. These behaviors were still maintained as the ZnGa_2_O_4_ NWs were exposed to below-band-gap irradiation. In contrast, upon exposure to a 254 nm UV light, the current drastically increased. With decreasing oxygen pressure or increasing temperature, the photocurrent also increased. The authors demonstrated that the surface-related processed (especially oxygen chemisorptions) had significant effects on the nanostructure photoelectronic properties [64].

#### 2.9.3 RuO_2_/TiO_2_ core/shell nanowire-based photodetectors

Chou *et al*. reported the fabrication of RuO_2_/TiO_2_ core/shell NWs by reactive sputtering, and investigated photoconductive properties [65]. These core-shell NWs had a square-like cross section and lengths of several micrometers. Upon exposure to a 256 nm UV light, the current of the RuO_2_/TiO_2_ core/shell structure rapidly increased, from 18.5 μA to 19.4 μA within 88 s, and then slowly increased to become saturated at 20 μA within 219 s during the first illumination. After the UV light was turned off, the current decreased from 20 μA to 19.4 μA within 52 s and then slowly recovered to the original value (under 385 nm illumination). The authors noted that the current decay rate was faster than the current increment rate. This was explained by a fact that water and oxygen molecules fast adsorbed on the surface via physiabsorption and chemiabsorption because of the large surface-to-volume ratio. The process resulted in a drastic drop in conductance as the UV light was turned off [65].

## 3. Conclusions and Outlook

In summary, this article provides a comprehensive review on recent advances in some important 1D metal-oxide nanostructures and their photodetector properties. Tables 2 and 3 are the up-to-date summaries of most important reports on photodetectors made of several metal-oxide nanostructures. Needless to say, due to the tremendous research efforts and the space limitations, this article is unable to cover all the exciting works reported in this field.

**Figure 11.**
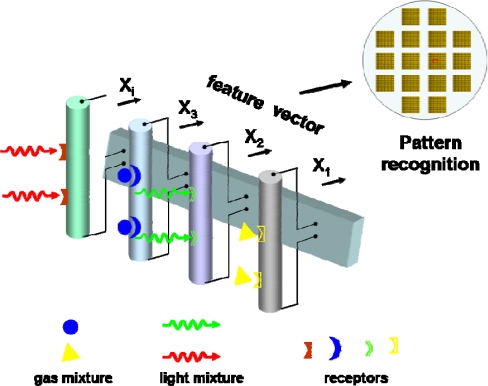
A sketch of a nanowire-based multi-functional detector [8].

It is noted that photoconductors based on 1D metal-oxide nanostructures have acquired fascinating achievements just in the last 10 years. However, the ways towards their practical applications are still endless and tortuous. This should inspire more research efforts to address the challenges that remain, as noted below:

1. Nanomaterial fabrication: As known, the material is the milestone of a device. The growth kinetics and thermodynamics involved in the synthesis of metal-oxide nanostructures are extremely complex, and presume different mechanisms under different growth conditions. Although comprehensive efforts have been made towards the synthesis of high-quality metal-oxide nanostructures, significant challenges still exist in their syntheses that include, but not limited to, reliable control of diameter, length, orientation, density, crystallization and hierarchical assembly.
2. Device fabrication: In the conventional “pick and place” method, 1D nanostructures fabricated by vapor synthesis process are first collected from substrates on which they were initially grown and then, dispersed randomly on an insulating substrate after being diluted in a solution. Sophisticated techniques such as photolithography, electron beam lithography or focused ion beam are required to make metallic contacts to the nanostructures. Out of question, this process is complicated, time-consuming and uneconomic, thus hampering the development of practical routes [21]. It still remains a grand challenge to construct a device via a simple and effective method. Furthermore, since the performance of devices critically depends on the quality of the Ohmic contacts between a nanostructure and the electrodes, the construction of reliable and stable contacts is an urgent task that deserves particular attention.
3. Sensitivity, selectivity and stability (3S): Next generation photodetectors will require significant improvements in sensitivity, selectivity and stability (3S) in order to meet the future demands in variety of fields. Though some research groups have successfully detected light using 1D metal oxide nanostructures, the selectivity and stability are still quite low.
4. Multi-functional detectors: The evolution of photodetectors goes in parallel with the development of microelectronics in which the architecture of photodetector elements is influenced by design trends in planar electronics. One of the major goals is to design nanodetectors that could be easily integrated into modern electronic fabrication technologies [8]. A possible avenue to differentiate the 1D nanostructure response maybe surface coating with chemical selective membrane, surface medication by specific functional groups, or combing multi-light and/or multi-gas sensing modules coupled with signal processing functions, acting as an “electronic nose” to differentiate in a more complex environment (Figure 11).

There is still plenty of room for the development of 1D metal-oxide nanostructures and their photodetector applications. We believe that future work in this direction should continue to focus on generating metal oxide nanostructures in a more controlled, predictable, reliable and simple way, and enhancing their photoconductor properties up to the level desirable in real industrial applications. The hopes are high that significant practical photodetector devices will soon arise due to the integration of 1D metal oxide nanostructures into conventional microelectronics.

## Figures and Tables

**Table 1.** Fundamental physical properties of some important metal-oxide semiconductors.

**Metal oxides**	**Crystal structures**	**Conductive type**	**Band gap/eV**	**Sensing light**

ZnO	Hexagonal	n	3.37	UV
SnO_2_	Tetragonal	n	3.6	UV
Cu_2_O	Cubic	p	2.17	Visible
β-Ga_2_O_3_	Monoclinic	n	4.2–4.9	UV
α-Fe_2_O_3_	Rhombohedral	n	2.1	Visible
In_2_O_3_	Cubic	n	3.6 (direct) 2.5 (indirect)	UV
CdO	Cubic	n	2.27 (direct) 0.55 (indirect)	Visible/IR
CeO_2_	Cubic	n	3.2	UV

**Table 2.** ZnO-based photodetectors.*

Nanostructures	Devices	Light of detection	Bias	Dark current Or conductance	Photocurrent Or conductance	Photocurrent and Dark current ratio	Rise time	Decay time	Ref.
NW	Resistor	390 nm; 6.3–40 mW/cm^2^	5	1–10 nA	100 μA	10^2^–10^5^	—	—	[9]
NW	Resistor	365nm; 0.3 mW/cm^2^	0–5	∼1 pA	∼250 nA	10^4^–10^6^	<1 s	<1 s	[12]
NW	Resistor	325 nm	0.5	∼0.05 μA	∼0.4 μA	—	—	—	[13]
NW	Resistor	340 nm	1	—	20 nA		170 s	300 s	[18]
NW	FET	365 nm; 0.47 mW/cm^2^	—	—	—	10^2^–10^6^	—	—	[19]
Al-ZnO NW	Resistor	400–800 nm	—	3.36×10^−4^ S/cm	6.67×10^−4^ S/cm	—	—	—	[20]
NW	Resistor	350 nm; 50 nW-48 mW/cm^2^	5	—	40 μA for 50 nW/cm^2^	10–10^5^	0.7 s	1.4 s	[21]
MT	Resistor	365 nm; 21700 μW/cm^2^	5	1.5 μA	0.085 mA (O_2_) 0.135 mA (air) 0.209 mA (N_2_) 0.201 mA (Ar)	—	2.9 s 5.9 s 28.4 s 45.8 s	100 s 638 s — —	[22]
NW	Resistor	633 nm; 0.2 W/cm^2^	2	13.1 nS	73.4 nS	—	—	—	[23]
ST ZnO NW	Resistor	365 nm; 30 μW/cm^2^	1	0.04 nA	60 nA	1500	0.6 s	6 s	[24]
Nanoneedle array	Resistor	365 nm; 16 μW/cm^2^	—	1.0×10^−4^ A	4.0×10^−4^ A	—	—	—	[25]
NW	Resistor	300–425 nm; 6 mW/cm^2^	10	20 nA	140 nA	—	—	—	[26]
NW array	Resistor	370 nm	5	70 μA	100 μA	—	0.4 ms	—	[27]
Co- ZnO NB	Resistor	370 nm	—	1.3 μA	110 μA	—	—	—	[28]
		630 nm			0.25 μA		500 s		
NR	Resistor	366 nm; 0.1 W/cm^2^	1	1200–4000 MΩ	20–500 MΩ	—	—	—	[29]
NW	Resistor	325 nm; 10 mW/cm^2^	0.5	—	—	—	—	—	[30]
NW	Resistor	254 nm; 7 W	—	0.08 μS	2 μS	5.8 (Vacuum) 66 (air)	16 s (V) 66 s (air)	188 s (V) 115 s (air)	[31]
ZnO NB PPAN-ZnO NB	Resistor	365 nm; 100 W	—	—	—	112% 9000%	—	—	[32]
NW	Resistor	254 nm (0.1 W/cm^2^) 366 nm (0.1 W/cm^2^)	0.25	100 nA	580 nA (254 nm) 700 nA (366 nm)	—	—	Tens of Seconds	[33]
NW array	Resistor	365 nm; 0.3 mW/cm^2^	5	1.35×10^−5^ A	2.0×10^−7^ A	150	—	—	[34]
NW array	Resistor	365 nm; 25 μW	20	0.15 μA	1.8 μA	—	—	—	[35]
NW arrays	Resistor	350 nm	5	—	—	11, 64	—	—	[36]
NW	Resistor	325 nm	5	—	0.35 nA	—	43.7 s (V) 4.6 s	—	[37]
Cu- ZnO NW	Resistor	365 nm; 5 mW/cm^2^	10	10 pA	100 nA	7000	—	—	[38]
		Visible; 10 mW/cm^2^			—	5000			
NW with CdTe QD	Resistor	450 nm; 10 mW/cm^2^	2	10^9^ Ω	12 nA	—	4.7 s	—	[39]
NR	Resistor	325 nm	2	1 nA	22 nA	—	3.7 s	63.6 s	[40]
NR	FET	254 nm	0.2	—	2.4 μA	10^3^	—	30 min	[41]
NW	Resistor	380 nm	5	2.5 nA	4.5 nA	—	—	17.0 s	[42]
NB	FET	350 nm	6.5	—	—	—	—	—	[43]
NW	Resistor	365 nm	2	2 μA	15 μA	—	45 s	55 s	[44]
NF/NWs	Resistor	115–400 nm; 150 W	10	7.38 nA	38.62	—	—	—	[45]

* NW-nanowire; MT-microtube; ND-nanoneedle; NB-nanobelt; NR-nanorod; QD-quantum dot; NF- Nanoflasks; V-vacuum

**Table 3.** Metal oxide-based photodetectors.*

Metal oxides	Nanostructures	Devices	Light of detection	Bias (V)	Dark current or conductance	Photocurrent or conductance	Photocurrent and Dark current ratio	Rise time	Decay time	Ref.
SnO_2_	NW	Resistor	370 nm	—	37.5 kΩ	12.5 kΩ	—	—	—	[47]
	NW	FET	254 nm	0.05	0.66 nS	760 nS	10^3^	—	< 0.1 s	[46]
	NB	Resistor	254 nm; 10 mW	−5	0.4 nA	80 μA (air) 900 μA (vacuum)	10^5^ (air) 10^6^ (vacuum)	—	—	[66]
			532 nm; 0.1–35.1 mW		1.4 nA	57 nA	—	< 1 s	< 1 s	
	NW	Resistor	325 nm; 100 W/m^2^	0.1	30 nA (air)	210 nA (air) 1.2 μA (Vacuum)	—	—	—	[67]
	NW	Resistor	365 nm	—	200 nA	235 nA	—	—	—	[68]
	ZnO-functional SnO_2_ NW			—		300 nA	1.50	—	—	
Cu_2_O	NW	Resistor	488 nm	3	0.7 μS	4.3 μS	∼6	< 3 s	< 3 s	[48]
β-Ga_2_O_3_	NW	Resistor	254 nm; 7 w	8	Several pA (15 pA)	Several nA (10 nA)	∼1000	0.22 s	0.09s	[51]
	NW	Resistor	254 nm	20	∼26 pA	∼0.56–0.0095 nA (P_O2_ = 22–20000 Pa)	—	—	—	[53]
CdO	ND	Resistor	950 nm	0.01	13.3 nS	114.5 nS	8.6	—	—	[60]
In_2_O_3_	NW	FET	254 nm 365nm	0.3	—	290 nA (254 nm) 33nA (365nm)	—	10 s	—	[57]
CeO_2_	NW film	Resistor	254 nm; 7W	5	22.8 nA (air) 0.35 pA (H_2_O)	0.25 nA (air) 0.44 nA (H_2_O)	∼1000	300 s (air) 2 s (H_2_O)	—	[61]
α-Fe_2_O_3_	NB	Resistor	400–800 nm; 0.5 mW/cm^2^	0.8	10 nA	123 nA (490 nm)	11–12	20 ms	—	[56]
α-MoO_3_	NB	Resistor	400–700 nm; 10 W	0.1	—	0.22 μA	—	—	—	[69]
MnO_2_	Nanosheet film	Resistor	450 nm	—	—	—	—	—	—	[70]
ZnSnO_3_	NW	Resistor	UV light; Green laser	—	0.3 nA	162 nA (UV) 6.6 nA (green)	—	20 s	—	[63]
ZnGa_2_O_4_	NW	Resistor	UV light	30	8.5 PA	1 nA	—	—	—	[64]
RuO_2_/TiO_2_	Core/Shell NW	Resistor	256 nm	—	18.5 μA	19.4 μA	—	307 s	437 s	[65]

* NW-nanowire; NB-nanobelt; ND-nanoneedle; NS-nanosheet; FET-field-effect transistors
